# Epidemiology and outcomes of community-onset methicillin-susceptible *Staphylococcus aureus *bacteraemia in a university hospital in Singapore

**DOI:** 10.1186/1471-2334-8-14

**Published:** 2008-02-07

**Authors:** Jonathan Wei-Zhong Chia, Li-Yang Hsu, Louis Yi-Ann Chai, Paul Ananth Tambyah

**Affiliations:** 1 Department of Medicine, Yong Loo Lin School of Medicine, National University of Singapore, 5 Lower Kent Ridge Road, Singapore 119074, Singapore; 2 Department of Medicine, National University Hospital, 5 Lower Kent Ridge Road, Singapore 119074, Singapore

## Abstract

**Background:**

Methicillin-susceptible *Staphylococcus aureus *(MSSA) bacteraemia remains a condition associated with considerable morbidity and mortality worldwide. It is a common but little-studied problem outside of Europe and North America.

**Methods:**

A single-centre retrospective case series profiling all patients with community onset-MSSA bacteraemia presenting between March 2005 and February 2006 to a tertiary acute-care university hospital in Singapore. In addition to epidemiological and clinical data collection, risk factors for complicated bacteremia and attributable mortality were analysed.

**Results:**

A total of 100 patients met the case definition. Patients were more likely to be male (65%) and below 65 years of age (69%). Seventeen patients were intravenous drug abusers, while 38 had diabetes mellitus. There were 18 cases of endocarditis, with 11 occurring in intravenous buprenorphine abusers. Attributable mortality was 11%, and 46% of patients developed complicated bacteremia. On multivariate analysis, age > 65 years and presence of chronic pulmonary disease were the only significant risk factors for the former, while valvular heart disease was a significant risk factor for the latter.

**Conclusion:**

MSSA bacteraemia is associated with a significant risk of serious complications in Singapore. Other Asian cities should be alert to the risk factors for adverse outcomes for this important cause of morbidity and mortality.

## Background

*Staphylococcus aureus *bacteraemia remains a common cause of mortality and morbidity both in spite of and as a consequence of medical advances. Although the mortality rate has declined in some countries as a result of improved quality of care [1], the overall prevalence has increased in line with increasing use of intravascular devices and an expanding "at-risk" population [1,2].

The focus in recent years has been on infections caused by methicillin-resistant *S. aureus *(MRSA), especially with regards to its clinical and economic impact in comparison with methicillin-susceptible *S. aureus *(MSSA) [3,4]. The rapid rise of infections caused by community-associated MRSA (CA-MRSA) has further directed attention towards MRSA [5,6]. Nevertheless, CA-MRSA is still relatively rare on a global basis, whereas MSSA is a more common cause of bacteraemia than MRSA in most parts of the world. The incidence of MSSA bacteraemias is also not significantly affected by the slew of infection control measures set in place in most health care institutions.

Previous landmark studies had described the epidemiology of MSSA bacteraemia and predictive factors for adverse outcomes of infection [2,7-9]. However, epidemiologic results are influenced by local factors, and community-onset MSSA (CO-MSSA) bacteraemia is little studied in Singapore and elsewhere outside of Europe and North America. This study describes the epidemiology of CO-MSSA bacteraemia and the risk factors for mortality and complicated infections in patients seen at a university hospital in Singapore, a modern Asian city.

## Methods

### Study Design

A retrospective chart review was conducted on all patients with CO-MSSA bacteraemia hospitalised at the National University Hospital – a 900-bed acute care tertiary academic hospital – between 1^st ^March 2005 and 28^th ^February 2006. The local ethics review board granted approval for the study.

### Subjects

The hospital microbiology laboratory generated a list of all MSSA-positive blood cultures for the study period and the patients' clinical charts were reviewed. A patient was defined as having CO-MSSA bacteraemia if MSSA-positive blood cultures had been drawn < 48 hours after hospitalisation or if he/she remained symptomatic with no other cause of infection found in the event that blood cultures were positive > 48 hours post-hospitalisation.

Patients were further classified into either community-acquired or healthcare-associated cases based on the criteria proposed by Friedman and co-workers [10]. Patients hospitalized for recurrent MSSA bacteremia or who had polymicrobial bacteremia were excluded from the study.

### Chart Review

A single investigator reviewed the patients' medical records, collating epidemiologic and clinical data in a designated database. We used the diagnoses recorded by the attending physicians, with the following exceptions: pneumonia was defined by new pulmonary infiltrates on a chest radiograph and isolation of MSSA from purulent sputum (with leukocytes but no epithelial cells seen on microscopy); endocarditis was defined strictly according to the Duke criteria [9]; and urinary tract infection was defined by a leukocyte count of ≥ 50 leukocytes/mm^3 ^of urine and a pure culture of MSSA yielding > 10^5 ^cfu/ml in patients with the appropriate clinical presentation.

Appropriate empirical therapy was defined as the empiric administration of antibiotics upon hospitalization to which the consequent MSSA isolate was susceptible.

Suboptimal therapy was defined by any one of the following: failure to initiate effective antistaphylococcal therapy (cloxacillin or cefazolin; vancomycin if the patient was allergic to penicillin) within 24 hours of a positive blood culture result; suboptimal dosages of antibiotics (< 4 g per day of cloxacillin or < 3 g per day of cefazolin; or their equivalent paediatric dosages where appropriate); antibiotic duration of < 10 days for uncomplicated bacteraemia and < 28 days for endocarditis, bone, joint or implant infections; and failure to remove accessible foci of infection.

### Outcomes Measured

The primary outcome measured was complicated bacteraemia. This was defined as the presence of either (1) attributable mortality – this included all patients who died with persistent signs and symptoms of infection in the absence of another explanation for death, (2) complicated infection at hospitalisation – new infection at a site distant from the primary focus caused either by haematogenous seeding or direct extension of infection, or (3) recurrent infection within a 12-week follow-up period. This is similar to the primary endpoint used by Fowler and co-workers with the exception that embolic stroke was not included within this definition [7]. Patients with more than one factor listed above were only counted once.

Secondary outcomes measured were attributable mortality and a composite variable comprising of complicated and/or recurrent infection.

### Statistics

Intercooled Stata (version 9.2) was used for statistical calculations. Dichotomous variables were analysed with the χ^2 ^test or Fisher's exact test as appropriate, and continuous variables were analysed with Student's *t *test. Univariate analyses of the association between individual variables and outcomes were performed using logistic regression. Variables with a *P *value of ≤ 0.20 on univariate analysis were subsequently included in the corresponding step-wise multivariate analysis. A *P *value of ≤ 0.05 was considered statistically significant.

## Results

Of 126 patients with blood cultures positive for MSSA in the study period, 100 fulfilled the criteria for CO-MSSA bacteraemia. Forty-eight (48%) fulfilled Friedman's criteria for healthcare-associated infection – the remainder were community-acquired [10]. The median duration of symptoms prior to hospitalization was four (range: 1 – 12) days. The details of the cases are listed in Table 1. Thirty-one patients were above 65 years in age. Twenty-six patients had central venous catheters – inserted for the purposes of either outpatient hemodialysis (92.3%) or chemotherapy (7.7%). One patient (1%) each had HIV infection and liver cirrhosis. All 17 intravenous drug users (IVDUs) reported abusing buprenorphine – 12 (70.6%) and 5 (29.4%) were of Malay and Indian ethnicity respectively. Fourteen (82.4%) IVDUs were male. All survivors had been followed-up at the hospital outpatient clinics for at least 12 weeks.

**Table 1:** Descriptive characteristics of 100 patients with CO-MSSA bacteraemia

Characteristic	Value^1^
Age, years	49.7 ± 21.6

Gender, male	65

Ethnicity	
- Chinese	51
- Malay	34
- Indian	12
- Others	3

Co-morbidities	
- Diabetes mellitus	38
- Dialysis-dependent renal failure	28
- Presence of malignancy^2^	14
- Chronic pulmonary disease^3^	10
- Valvular heart disease	4

Other characteristics	
- Intravenous drug abuse	17
- Presence of intravenous catheter^4^	26
- Previous hospitalisation^5^	53

Appropriate empiric therapy	94
Optimal antibiotic therapy	68

^1^Data are number or mean ± standard deviation

^2 ^Includes both solid and haematologic malignancies

^3 ^Includes chronic obstructive pulmonary disease, pulmonary fibrosis, and bronchiectasis

^4 ^Central venous catheters only

^5 ^Within 12 months of current hospitalisation

Although all but six patients received appropriate empiric antibiotics upon hospitalization, optimal therapy as defined previously was not received by 32 patients. This included suboptimal antibiotics for 27 patients, and inappropriate dosage/delivery in 5 – oral cloxacillin at a dosage of 2 g per day had been used. The suboptimal antibiotics used included vancomycin in 11 (40.7%) patients who were not allergic to penicillin, ceftriaxone in eight (29.6%), amoxicillin/clavulanate in two (7.4%), levofloxacin in one (3.7%), and combinations of the above in the remaining five (18.5%) patients. All eradicable foci of infection, including central lines, abscesses, and infected implants and joints were removed and/or drained appropriately.

Patient outcomes, including causes of death other than CO-MSSA bacteraemia and breakdown of complicated infections, are listed in Table 2. Of the 46 patients with the primary outcome of complicated bacteraemia, seven (15.2%) were re-admitted for recurrent MSSA infection, whereas 33 (71.7%) had complicated infection. Two of eleven patients who died of MSSA bacteraemia and three of seven patients re-admitted for recurrent MSSA infection met the definition of having complicated infections at first presentation. Eleven of 18 patients with infective endocarditis were IVDU's. The crude mortality for patients with CO-MSSA bacteraemia was 18%. Median duration of stay among survivors was 11 days, with an inter-quartile range of 20.5 days.

**Table 2:** Outcomes of 100 patients with community-onset methicillin-susceptible *Staphylococcus aureus *bacteraemia

Outcome	Number (%)
• No complications from bacteraemia	54
◦ Other cause mortality	7 (13.0)
▪ Acute myocardial infarction	3 (42.8)
▪ Liver failure	2 (28.6)
▪ Leukaemia/Lymphoma	2 (28.6)

• Complicated bacteraemia	46
◦ Attributable mortality	11 (23.9)
▪ Pneumonia	5 (45.4)
▪ No primary site	4 (36.4)
▪ Soft tissue infection	1 (9.1)
▪ Infective endocarditis	1 (9.1)
◦ Recurrent infection within 12 weeks	7 (15.2)
▪ Intravascular infection	3 (42.8)
▪ Soft tissue infection	2 (28.6)
▪ Infective endocarditis	2 (28.6)
◦ Complicated infection at initial hospitalisation^1^	33 (71.7)
▪ Infective endocarditis	18 (54.5)
▪ Soft tissue infection/necrotizing fasciitis	6 (18.2)
▪ Psoas/paravertebral abscess	6 (18.2)
▪ Vertebral osteomyelitis/disciitis	3 (9.1)
▪ Epidural abscess	3 (9.1)
▪ Other osteomyelitis	1 (3.0)
▪ Liver abscess	1 (3.0)
▪ Brain abscess	1 (3.0)
▪ Pulmonary abscess/Pneumonia	1 (3.0)

^1 ^Some patients have multiple sites of infection. However, each case is only counted once

Results of univariate analysis of patient characteristics with outcomes are shown in Table 3. Because suboptimal therapy has no impact on the presence or absence of complicated infections at presentation, the association of this variable with the primary outcome was not calculated. Patients with valvular heart disease or who abused intravenous buprenorphine were at higher risk of complicated bacteraemia. Patients above 65 years of age and patients with chronic pulmonary disease were more likely to die as a consequence of CO-MSSA bacteraemia. Patients with central intravenous catheters, who abused intravenous buprenorphine, underwent hemodialysis, or who were of non-Chinese ethnicity were more likely to develop a complicated infection and/or recurrent infection.

**Table 3:** Impact of patient characteristics on outcomes measured

Characteristic	Complicated bacteraemia		Attributable mortality		Complicated and/or recurrent infection	
	
	Odds ratio [CI]	*p*-value	Odds ratio [CI]	*p-value*	Odds ratio [CI]	*p-value*
Demographics:						
Age > 65 years	1.39 [0.59–3.24]	0.45	13.70 [2.75–68.29]	< 0.01	0.48 [0.19–1.22]	0.12
Male gender	0.82 [0.36–1.88]	0.64	1.07 [0.29–3.94]	0.92	1.01 [0.43–2.36]	0.98
Ethnicity	1.59 [0.95–2.64]	0.08	1.10 [0.52–2.36]	0.80	1.73 [1.03–2.90]	0.04
Healthcare-associated infection	0.51 [0.23–1.14]	0.10	1.34 [0.38–4.72]	0.65	0.43 [0.19–1.00]	0.05

Co-morbidities:						
Diabetes mellitus	0.55 [0.24–1.25]	0.15	0.58 [0.14–2.33]	0.44	0.68 [0.29–1.60]	0.38
Dialysis-dependent renal failure	0.71 [0.32–1.57]	0.40	3.27 [0.81–13.13]	0.10	0.36 [0.15–0.84]	0.02
Malignancy	0.42 [0.12–1.44]	0.17	2.66 [0.61–11.56]	0.19	0.24 [0.05–1.15]	0.08
Chronic pulmonary disease	1.88 [0.50–7.10]	0.36	14.00 [3.15–62.17]	< 0.01	0.17 [0.02–1.37]	0.10
Valvular heart disease	5.47 [1.10–27.25]	0.04	2.25 [0.41–12.26]	0.35	2.85 [0.75–10.88]	0.12

Other risk factors:						
Intravenous drug abuse	4.92 [1.48–16.41]	< 0.01	-*	-*	7.99 [2.37–26.98]	< 0.01
Presence of intravenous catheter	0.53 [0.21–1.34]	0.18	2.70 [0.75–9.74]	0.14	0.31 [0.11–0.92]	0.03
Previous hospitalization	0.68 [0.31–1.50]	0.34	2.61 [0.65–10.47]	0.18	0.54 [0.24–1.22]	0.14
Inappropriate empiric therapy	0.57 [0.10–3.25]	0.55	4.72 [0.75–29.5]	0.10	-*	-*
Suboptimal therapy	Not applicable	-	2.91 [0.82–10.37]	0.10	Not applicable	-

* This calculation predicted failure in a significant way. No intravenous drug abuser died from *S. aureus *bacteremia, and none of the patients with complicated and/or recurrent infections received inappropriate empiric therapy.

On multivariate analysis, only the presence of valvular heart disease (OR, 6.85; 95%CI, 1.21 – 38.72; *p *= 0.03) was independently associated with complicated bacteraemia. Age > 65 years (OR, 13.66; 95%CI, 1.74 – 107.39; *p *= 0.01) and chronic pulmonary disease (OR, 9.83; 95%CI, 1.56 – 62.05; *p *= 0.02) were independently associated with attributable mortality. Inappropriate empiric therapy (*p *= 0.13) and suboptimal therapy (*p *= 0.20) did not appear to have any independent impact on attributable mortality. Intravenous drug abuse (OR, 5.54; 95%CI, 1.30 – 23.62; *p *= 0.02) was independently associated with the development of complicated and/or recurrent infection.

## Discussion

This is the first attempt to characterise CO-MSSA bacteraemias in Singapore. MSSA remains one of most common causes of community-onset bacteraemias worldwide, as evidenced by reports from Laos and Africa [11,12], although there are no specific analyses of MSSA bacteraemia outside of Europe or North America. It is important to note that 48% of the cases would fulfill criteria for healthcare-associated infections [10]. However, this had no independent impact on the outcomes measured. Because up to 30% of the general population is colonized by *S. aureus*, it is difficult to determine if patients with healthcare-associated MSSA infections by definition are actually infected by their own endogenous MSSA rather than a transmitted healthcare-associated strain.

It is interesting to note that suboptimal therapy as defined by Jensen and coworkers was not associated with mortality in our series [8]. It is possible that this is due to the smaller sample size, but the fact that all eradicable foci were removed from our patients and that 94% of patients received empiric antibiotics active against MSSA probably also contributed to the relative success of using suboptimal therapy.

One point of contention is the narrow definition of suboptimal therapy itself. Antibiotics such as amoxicillin-clavulanate, levofloxacin, and ceftriaxone have *in-vitro *activity against MSSA, but they are not recommended on any published guidelines for the treatment of MSSA bacteraemia. Although vancomycin is demonstrably inferior to cloxacillin in the treatment of MSSA endocarditis [13], it is less clear if this remains the case for the treatment of uncomplicated bacteraemia. However, further analyses (data not shown here) re-classifying these antibiotics as "optimal" for the treatment of MSSA bacteraemia did not change the overall result that suboptimal therapy had no impact on attributable mortality.

The skewed distribution of CO-MSSA bacteraemia cases among the ethnic groups as compared with the national demographic is partly contributed by the ethnic distribution of the IVDUs [14]. A previous article had reported that half of the intravenous buprenorphine abusers in Singapore were of non-Chinese ethnicity, although these minorities make up only 25% of Singapore's population [15]. The issue of health differentials in Singapore's different ethnic groups has been highlighted for chronic diseases [16], and now appears to be an issue for acute infections as well.

The high incidence of endocarditis, much higher than in the studies of Fowler and Jensen despite < 50% of local patients undergoing echocardiography is a consequence of the relatively large proportion of IVDUs in our series. This highlights a separate social issue of buprenorphine abuse in Singapore – itself a consequence of loose regulatory control over the drug in conjunction with stringent and tightly enforced regulations on other intravenous opiates – which at its height was responsible for more than 50 deaths over a two-year period [14]. New regulatory and rehabilitation programs implemented since August 2006 should result in the decline of such cases over time [14].

## Conclusion

As European and North American investigators have found, community-onset *S.aureus *bacteraemia is associated with a significant morbidity and mortality, especially in older individuals with co-morbidities in Singapore. This highlights the need for continued research into treatment advances and preventive strategies for *S. aureus *as a whole, and not just for MRSA, in the rest of the world as well.

## Competing interests

The author(s) declare that they have no competing interests.

## Authors' contributions

JWZC collected the patient data and wrote the first draft of the manuscript. LYH performed the statistical analyses and wrote the submitted version of the manuscript. LYAC assisted with the designing of the study and proofreading of the manuscript. PAT developed the study design and provided overall support and proofreading of the manuscript. All authors have read and approved the final manuscript.

## Pre-publication history

The pre-publication history for this paper can be accessed here:


